# Social and Anxiety‐Like Behaviors Are Affected in Juvenile Mice With *Gli2^+/−^* but Not *Gli3^+/𝛥699^* Genetic Modifications

**DOI:** 10.1002/brb3.71503

**Published:** 2026-05-31

**Authors:** Thomas Niepsuj, Rithika Nurani, Leykza Carreras‐Simons, Wade Bushman, Joan S. Jorgensen, Walid A. Farhat, Anthony P. Auger

**Affiliations:** ^1^ Department of Psychology University of Wisconsin Madison Wisconsin USA; ^2^ Division of Pediatric Urology, School of Medicine and Public Health University of Wisconsin Madison Wisconsin USA; ^3^ Department of Comparative Biosciences, School of Veterinary Medicine University of Wisconsin Madison Wisconsin USA

**Keywords:** anxiety behavior, GLI, juvenile social behavior, variations in sex characteristics

## Abstract

**Introduction:**

*Gli2^+/−^*, *Gli3^+/𝛥699^*, and *Gli2^+/−^*; *Gli3^+/𝛥699^* mutant mice, novel models for variations in sex characteristics (VSCs), were used to investigate the impact of GLI2 and GLI3 modulation on the relationship between the development of anxiety‐like behavior, social behavior, and peripheral morphology.

**Aim:**

Uncover how altered GLI profiles modulate urogenital, neuroendocrine, and behavioral systems.

**Hypothesis:**

Increased disruption of the GLI2/GLI3 regulatory axis will display increases in the severity of urogenital dysmorphogenesis and concurrent alterations in juvenile social and anxiety‐like behaviors.

**Methods:**

Male and female mice were tested in social interaction and open field testing at postnatal days (PNDs) 24–26 and 26–29, respectively. Following behavioral testing, anogenital distance (AGD), plasma steroids: testosterone and corticosterone, and the volume of the sexually dimorphic nucleus (SDN) were taken and analyzed.

**Results:**

Significant differences in social and anxiety‐like behaviors among genotypes were documented. Female double heterozygote *Gli2^+/−^*; *Gli3^+/𝛥699^* mice showed reduced social behavior and increased anxiety‐like behaviors. *Gli2^+/−^* mice exhibited sex‐specific changes in anxiety‐like behaviors. Conversely, no behavioral differences were observed in *Gli3^+/𝛥699^* mice. *Gli2^+/−^* mice and female *Gli2^+/−^*; *Gli3^+/𝛥699^* mice showed reduced AGD. No genotypic variations were observed in SDN volume or plasma steroids.

**Conclusion:**

These data suggest that morphogens that are involved in peripheral variations in sexual characteristics may also be involved in regulating anxiety‐like and social development. This not only underscores the importance of mouse models in understanding genital variations and their relationship to juvenile behavior but also may be used to inform clinical approaches to those with VSCs with increased mental health concerns.

## Graphical abstract

Across *Gli3* ^+/Δ699^, *Gli2^+/−^*, and combined *Gli*2^+/−^;*Gli3^+/Δ699^* mice (males and females, with wild‐type controls), GLI2 and GLI3 differentially regulate anogenital distance and juvenile behavior, with amplified effects under combined disruption, whereas circulating steroids and sexually dimorphic nucleus area remain unchanged. Created in BioRender. Niepsuj, T. (2026) https://BioRender.com/arcw1wy

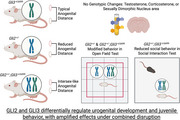

## 1 Introduction

Instrumental to mammalian development, GLI proteins function as key transcriptional effectors within the Sonic, Indian, and Desert Hedgehog (HH) signaling pathways (Niewiadomski et al. 2019; Hui and Angers 2011). Mechanistically, the GLI family of zinc finger transcription factors are activated through HH ligands binding to the receptor Patched (PTCH1), which in turn relieves inhibition of the signal transducer, Smoothened (SMO), across the primary cilium (Goetz and Anderson 2010). Through the coordinated activation and repression of downstream target genes, these pathways orchestrate critical aspects of developmental patterning, including organogenesis, urogenital morphogenesis (Miyagawa et al. 2011; Shehata et al. 2011), neuronal migration (Taroc et al. 2020), the formation of diverse neural structures, and brain development (Franz 1994; Palma and Ruiz i Altaba 2004; Ruiz i Altaba et al. 2002; Haddad‐Tóvolli et al. 2015; Bertrand and Dahmane 2006; Cai et al. 2023); underscoring their essential role in maintaining tissue patterning and homeostasis.

Perturbations in HH–GLI signaling disrupt core developmental programs and have been strongly implicated in a spectrum of human developmental anomalies and pathological phenotypes (Villavicencio et al. 2000). These changes underlie diverse congenital anomalies, including hypospadias (Saraç et al. 2021) and gonadal dysgenesis (Umehara et al. 2000), both clinically classified as variations in sex characteristics (VSCs) (Nieuwenhuis and Hui 2004). Importantly, individuals with VSCs exhibit increased incidence of neurodevelopmental, anxiety, and affective disorders (Butwicka et al. 2014; Jin et al. 2022; Zeeman and Aranda 2020; Falhammar et al. 2018; Rosenwohl‐Mack et al. 2020). Given this cooccurrence, disruptions of critical HH–GLI signaling pathways to central and peripheral systems might be responsible for both mental health and urogenital outcomes for those with VSCs; with defective HH–GLI transcriptional regulation during organogenesis altering morphogen gradients and cell fate specification. Notably, the underlying mechanisms to mental health disparities in this community remain elusive due to social confounders and incomplete understanding of how peripheral developmental signals interface with behavioral circuitry.

Rodent models allow dissection of this developmental coupling. For example, Desert HH‐null mice, a model for VSCs with disrupted urogenital morphogenesis, display increased anxiety‐ and depression‐like behaviors (Umehara et al. 2006). Such phenotypes suggest that HH–GLI‐dependent transcriptional patterning contributes to both genital and neural development through shared embryonic field interactions. Yet the specific contributions of GLI activator–repressor balance on behavioral phenotypes remain uncharacterized and are the focus of our present study. Hence, we explore the connection between behavioral development and VSCs, laying the groundwork for understanding the role of GLI proteins in disordered states.

To interrogate these mechanisms, this study employs *Gli2^+/−^*, *Gli3^+/𝛥699^* and *Gli2^+/−^*;*Gli3^+/𝛥699^* compound mutants, which model graded reductions in *Gli2* and *Gli3* transcriptional output and mirror human VSC phenotypes, such as hypospadias and cryptorchidism (He et al. 2016; Kothandapani et al. 2020; Yadav et al. 2023). By integrating behavioral and morphometric analyses across sexes (Bowman et al. 2025; Quinn and Koopman 2012), we aim to uncover how altered GLI profiles modulate juvenile behavioral circuits—which are governed by neural developmental trajectories—linking GLI‐mediated development of urogenital, neuroendocrine, and behavioral systems. Although GLI‐mediated processes most certainly work during distinctive spatiotemporal points, we chose to investigate the juvenile period, as it is a window of maturation where behavioral changes can give insights into mental health vulnerability (Uhlhaas et al. 2023). We hypothesize that increasing disruption of the GLI2/GLI3 regulatory axis will display increases in the severity of urogenital dysmorphogenesis and concurrent alterations in social and anxiety‐like behaviors that will be distinct during the juvenile stage.

## 2 Methods

### 2.1 Animals

All mice were derived from a CD‐1 background and mated as described previously (He et al. 2016). *Gli2^+/−^* mice have a targeted deletion of the DNA‐binding zinc finger motif in the *Gli2* gene, thus decreasing transcriptional activation and regulation of GLI2 responsive genes (Mo et al. 2001). *Gli3^+/𝛥699^* mice encode a GLI3 protein with a C‐terminal truncation due to a deletion 3’ of the zinc finger motif in the *Gli3* gene (Böse et al. 2002). This change renders the repressor form of the protein to be constitutively active, thus enhancing the GLI3 protein repressor function.

The mice were housed in aseptic, disposable, ventilated Innovive cages that are irradiated. Cages have the following specifications: capacity: suitable for up to five mice, floor space: 522.6 cm^2^, dimensions: 37.3 cm × 23.4 cm × 14.0 cm, height/headroom: 5 in./12.7 cm. They receive autoclaved 1/8″ corn cob bedding and Enviro‐Dri nesting material for enrichment. Irradiated igloos are provided for breeding cages. The mice are fed either 2920× or 2919, Irradiated Harlan Teklad Global Soy Protein‐Free Extruded Rodent Diets and given acidified water. The facility maintains a temperature of 22°C (±2°C) with 30%–70% humidity and operates on a 12‐h light/12‐h dark cycle (0700‐1900). Access to the facility is restricted, keeping outside noise low and rodent disturbances to a minimum. Post‐weaning cohorts are kept with same‐sex cage mates, with a maximum of five mice per cage. All husbandry protocols were kept in compliance with our Biomedical Research Model Services facility.

**FIGURE 1:**
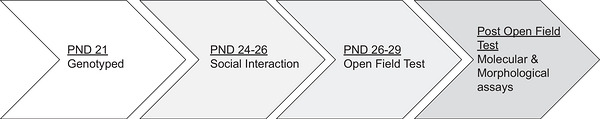
Experimental timeline. Timeline of animal protocols and procedures. PND, postnatal day.

Genotype and sex were confirmed on postnatal day 21 (PND 21), our weaning age, by polymerase chain reaction (PCR) of ear‐notch DNA, using primers specific for *Gli2^+/−^* and *Gli3^+/𝛥699^*, along with Sry primers to sex males (Table 1). All protocols and procedures involving animals were reviewed and approved by the Institutional Animal Care and Use Committee (IACUC) of the University of Wisconsin under protocol ID: M006295‐A01. The timeline of animal protocols and procedures is summarized in Figure 1.

**TABLE 1:** Polymerase chain reaction (PCR) genes of interest and primer sequences.

Gene	Genotype	Forward primer	Reverse primer	Reporter 1
*Gli2*	*Gli2^+/−^*	TGCGTCCAAGTTACTTTTATACTGCAA	CATTCTCAGTATTGTTTTGCCAAGTTCT	CCATCAGAAGCTGACTCTAG
*Gli3*	*Gli3^+/𝛥699^*	ACTGAAGGCTCTTTACTATTGCTTTATGATAATGTTTC	GTGGAAATGGTCGAGTCCATGAT	CAAATTAAGGGCCAGCT
*Sry*	Y	GCTTCAGTAATCTCAGCACCTAGAA	CACATTGGCATGATAGCTCCAAATT	ATGCTGACAAATATCC

*Note*: Primer sequences used for polymerase chain reaction genotyping.

### 2.2 Behavioral Testing

Male and female *Gli2^+/−^*, *Gli3^+/𝛥699^*, and double heterozygous *Gli2^+/−^*; *Gli3^+/𝛥699^* mice were tested along with wild type (WT) controls on each day of behavioral testing. All tests were conducted during the last 4 h of the light phase, just prior to the normal wake cycle.

Behavioral testing was conducted as genotypes were validated. Due to the difficulty in obtaining genotypes of interest, testing was performed in cohorts across several months. To account for potential variations due to different test days or seasonal changes, each day's testing included rodents from all experimental groups. Only testing days with all four groups (WT male/female and mutant male/female) represented were included in the final data set. WT animals were pooled by sex across days, but only from days when the corresponding mutant genotype was tested. Rodents were selected for testing in a random order, and after completing a test, they were placed in a holding cage separate from their cage mates. Once all rodents from the same cage had completed testing, they were reunited in their home cage. All testing was done by the same researcher to keep the testing environment consistent.

Two behavioral tests were used to assess the impact of genotype on rodent juvenile behavior. First, social interaction testing served as an assay for rodent behaviors indicative of sociability (Panksepp et al. 2007). Specifically, increased time engaging in social investigation suggests a higher degree of sociability in rodents, while decreased time engaging in social interactions could suggest social anhedonia. Second, the open field test served as an assay for evaluating locomotor and anxiety‐like behaviors in rodents (Seibenhener and Wooten 2015), where subjects are introduced to a novel arena and their exploratory activity is observed. Rodents spending more time in the center of the arena and exposing themselves to potentially high‐risk situations are seen as exhibiting a less anxious‐like phenotype. In contrast, a rodent exhibiting less exploratory behavior, lingering in the periphery, and remaining stationary near the wall‐floor junction is seen as displaying a more anxious‐like phenotype. Social interaction and open field assays were selected to provide an initial, broad assessment of social behavior and general locomotor/exploratory activity to lay the novel groundwork of behavioral investigation in these models.

#### 2.2.1 Social Interaction Testing

On PND 24–26, mice were subjected to social interaction testing. Prior to testing, each mouse was single housed for 24 h in a clean, dimly lit, red light (35 lx) cage. Five minutes before testing, the top from a test mouse cage is replaced with transparent Plexiglas and then the cage is positioned under a camera and left alone for an acclimation period of 5 min. Following isolation, a randomly selected, age‐matched, same sex, experimentally naive C57BL/6J stimulus mouse was placed into the test mouse's cage. C57BL/6J stimulus mice were used due to their inbred background, reducing behavioral variability and allowing for more reliable measurement of the test mice's social behaviors. Behavior monitoring was immediately recorded for 5 min. Social investigation was scored by a human observer to capture overall social interaction patterns between our mice. This was quantified by measuring the duration of time the test mouse spent sniffing (direct nose‐to‐body contact or test mouse positioning its nose within approximately 1 cm of the stimulus mouse), allo‐grooming, or following the stimulus mouse within one body length. Additionally, rudimentary locomotor social behavior was assessed by measuring “jerk and run” behaviors, which is a fast hopping or twitching behavior in the juvenile mouse when alongside another mouse, and then followed by erratic running behavior that is non‐goal directed (Wolff 1981). No notable aggressive, pouncing, or pinning social behaviors were observed in these mice, as we have assessed previously in rats (Auger and Olsen 2009; de Faria Oliveira et al. 2024).

#### 2.2.2 Open Field Testing

On PND 26–29, mice were evaluated in the open field test. Under normal lighting (450 lx), mice were brought to the arena room for 30 min for room acclimation. During acclimation, four polycarbonate open field arenas (41 cm × 41 cm) (Wachi et al. 2016) were sprayed using hydrogen peroxide solution, let to sit for 30 s, and wiped dry. Additionally, Fusion 6.5 SuperFlex from Omnitech Electronic software (Columbus, Ohio) was set up for data collection (Anchan et al. 2014). Following acclimation, all four arenas were wiped down with a paper towel sprayed with 70% ethanol and let dry. Once dry, one mouse was placed in the center of each arena, and a top cover was put on the arena. Open field arenas were spaced six inches apart and covered with lids to minimize external interference. Given the room size (approximately 110 sq ft.) and the spatial separation of the arenas, it is unlikely that vocalizations between mice influenced behavior during testing. The observer then quietly leaves the arena space, and mice are left to explore the novel environment for 10 min. Each arena is equipped with an array of horizontal and vertical infrared beams. Beam interruptions automatically detect overall movement as well as record time spent in the central 25% versus peripheral 75% of each arena. Additional documented behaviors included distance traveled, time spent resting, vertical activity, and non‐locomotive beam breaks; with specification indicating location of each behavior (i.e., center or periphery). Non‐locomotive beam breaks are more specifically when the same beam or set of beams are broken repeatedly without recording changes in distance, often occurring during grooming or head bobbing behavior. At the end of each test, rodents were placed back in their home cage, the arena was sprayed with 70% ethanol and let dry, and a new cohort of mice was tested.

### 2.3 Endocrine Assays

Following behavioral testing, mice were anesthetized via isoflurane inhalation in preparation for an axillary bleed. Blood was collected into EDTA‐treated tubes, kept on ice, and centrifuged at 20,000 × *g* for 15 min. All plasma samples were processed under sterile conditions with the top 200 µL of plasma being isolated and stored at −20°C until further analysis. Following blood collection animals are then euthanized by cervical dislocation in accordance with institutional ethical guidelines. Frozen plasma samples were submitted to Wisconsin National Primate Research Center Assay Services (Madison, Wisconsin) for steroid analysis. Corticosterone and testosterone were quantified, as biological markers for stress and androgen exposure, respectively, using high‐performance liquid chromatography–tandem mass spectrometry using a Sciex 6500+ Mass Spectrometer.

#### 2.3.1 Perfusions and Histological Analysis of the Sexually Dimorphic Nucleus (SDN)

A subset of mice was euthanized and perfused following behavioral experiments. At euthanasia, animals were anesthetized and perfused with 4% paraformaldehyde, and brains were harvested immediately. Brains were postfixed in 30% sucrose, embedded in optimal cutting temperature (OCT) blocks, and sectioned at 40 µm thick at −20°C for histological analysis. The sections were then stained with Hoechst 33342 to identify nuclear clusters. The SDN was localized using the Paxinos and Watson Rat Brain Atlas and Bloch and Gorski (1988) and analyzed as we have done previously (Auger et al. 2000). SDNs were then inspected by an experimenter unaware of groups under the DAPI filter set (excitation 330–385 nm, emission >420 nm) using an Olympus BX‐61 microscope. Areas of the SDN were then manually traced using Olympus MicroSuite (Soft Imaging Corp.) software, and areas were taken for statistical analysis.

#### 2.3.2 Anogenital Distance (AGD) Measurements

AGD measurements were done on euthanized animals. AGD is defined as the distance from the center of the anus to the tip of the penis or the opening of the vagina as an indicator of androgen exposure. The mice were placed in a supine position with the average length from three replicate measurements used for analysis.

#### 2.3.3 Data Analysis and Manuscript Preparation

All data were analyzed using GraphPad Prism 10 Software version 10.5.0 (673) for MacOS (GraphPad Software, San Diego, California). GraphPad Prism's Grubbs test was utilized to remove singular outliers, allowing no more than one outlier to be removed per group per data set. Data on *Gli2^+/−^* and *Gli3^+/𝛥699^* mice were analyzed using two‐way analysis of variance (ANOVA) with main effects examining genotypes and sex. When interactions between Genotype x Sex were detected, comparisons were followed by post hoc analysis using Tukey's tests. Data on female *Gli2^+/−^*; *Gli3^+/𝛥699^* mice were analyzed using the unpaired *t*‐test, with all data passing the Shapiro–Wilk normality test. Pearson correlations were used to evaluate the association between hormones, behavioral outcomes, neuroanatomy, and AGD in all data sets. All figures express mean ± SEM, and all statistical significance was set at a *p* value less than or equal to 0.05. The authors used ChatGPT (OpenAI, GPT‐5) for language editing and improving sentence structure and grammar. All data, analyses, and conclusions are the original work of the authors.

## 3 Results

### 3.1 *Gli2^±/−^* Behavior

**FIGURE 2:**
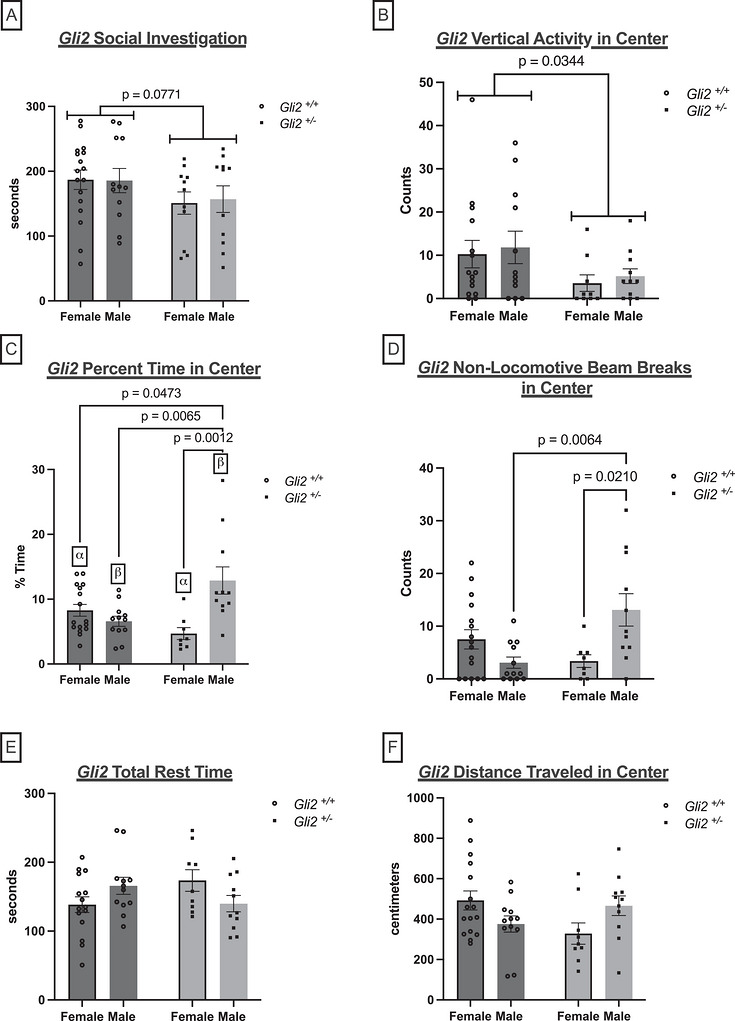
*Gli2^+/−^* behavior results. (A) Time spent sniffing, grooming, or following the stimulus mouse within one body length. (B) The number of breaks in vertical infrared beams indicating total vertical activity. (C) Percentage of time rodents spent in the center of the open field test. Increased time indicates reduced anxiety‐like behavior. [*α*] versus [*β*] represent statistically different groups. (D) The number of non‐locomotive beam break behaviors in the open field test, as defined as when the same infrared beam or set of infrared beams are broken repeatedly without changes in distance, often occurring during grooming or head bobbing behavior. (E) Total rest time in the open field test. (F) Total distance traveled in the center of the open field test.

Social interaction testing revealed a nonsignificant, but trending reduction, in social investigation (Figure 2A). No differences in “jerk and run” behavior were observed. Anxiety‐like behaviors were significantly different among *Gli2^+/−^* mice, with some sex‐specific differences. Two‐way ANOVA analysis of vertical activity in the center, a behavior associated with self‐motivated, nonsocial exploration, indicated a main effect of genotype (*p* = 0.034), signifying that *Gli2^+/−^* mice spent less time engaging in such behavior (Figure 2B). Two‐way ANOVA analysis of the percentage of time spent in the center of the arena indicated a significant interaction between genotype and sex (*p* = 0.0005) and a main effect of sex ([*α*] vs. [*β*], *p* = 0.0171). A Tukey post hoc comparison revealed multiple significant differences. Specifically, *Gli2^+/−^* males spent significantly more time in the center of the open field test (Figure 2C). Further analysis of behaviors in the open field test exposed differences in non‐locomotive beam break behavior in the center (Figure 2D), as defined as when the same beam or set of beams are broken repeatedly without recording changes in distance typically occur during grooming or head bobbing behavior. This phenotype follows a similar pattern to the percentage of time spent in the center of the arena; with two‐way ANOVA analysis indicating a significant interaction between genotype and sex (*p* = 0.0015). Post hoc analysis revealed *Gli2^+/−^* males spending significantly more time engaging in non‐locomotive beam break behaviors. Total rest time (Figure 2E) and distance traveled in the center (Figure 2F) also indicated a significant interaction between genotype and sex (*p* = 0.0222 and *p* = 0.0124, respectively). No differences in other behaviors during open field testing were observed.

### 3.2 *Gli3^+/𝛥699^* Behavior

**FIGURE 3:**
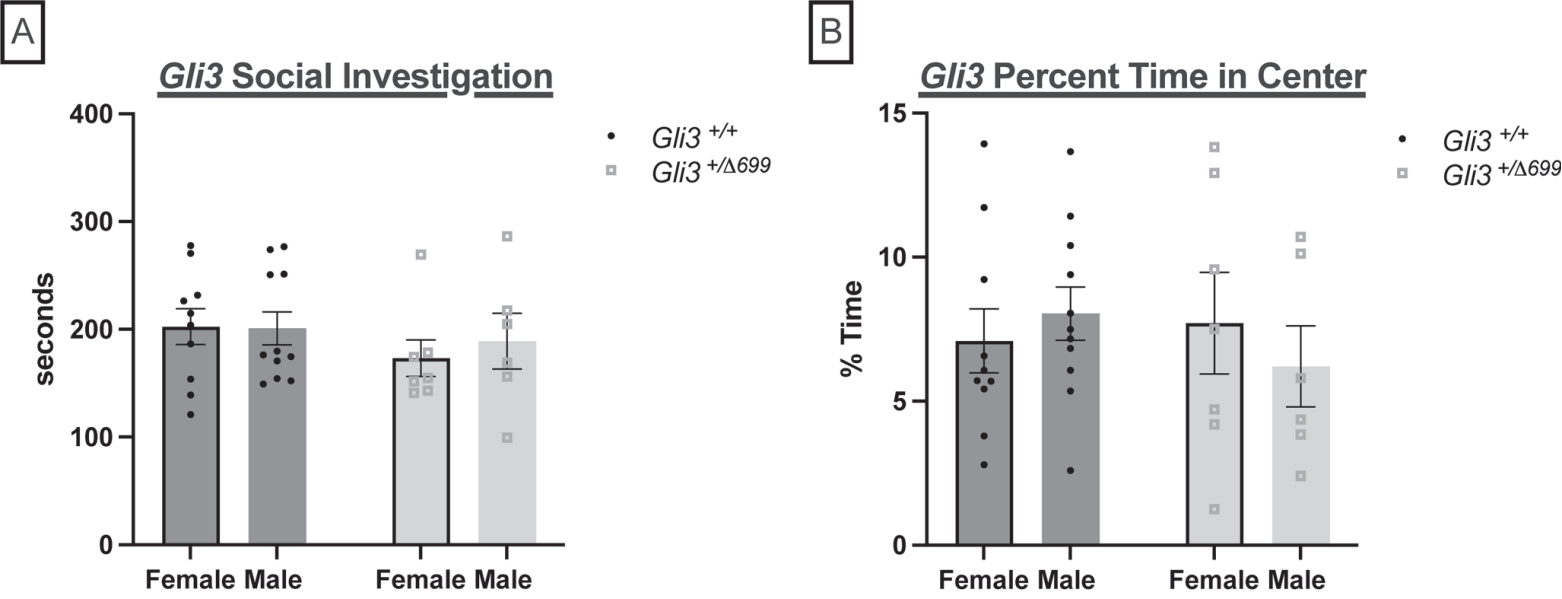
*Gli3^+/𝛥699^* behavior results. (A) Time spent sniffing, grooming, or following the stimulus mouse within one body length. (B) Percentage of time rodents spent in the center of the open field test. No differences were observed.

Across all recorded social investigation and anxiety‐like behaviors, no significant differences were found in *Gli3^+/𝛥699^* mice (not all data shown), indicating that *Gli3^+/𝛥699^* modification alone has no impact on juvenile social (Figure 3A) or anxiety‐like behavior (Figure 3B).

### 3.3 *Gli2^+/−^*; *Gli3^+/𝛥699^* Behavior

**FIGURE 4:**
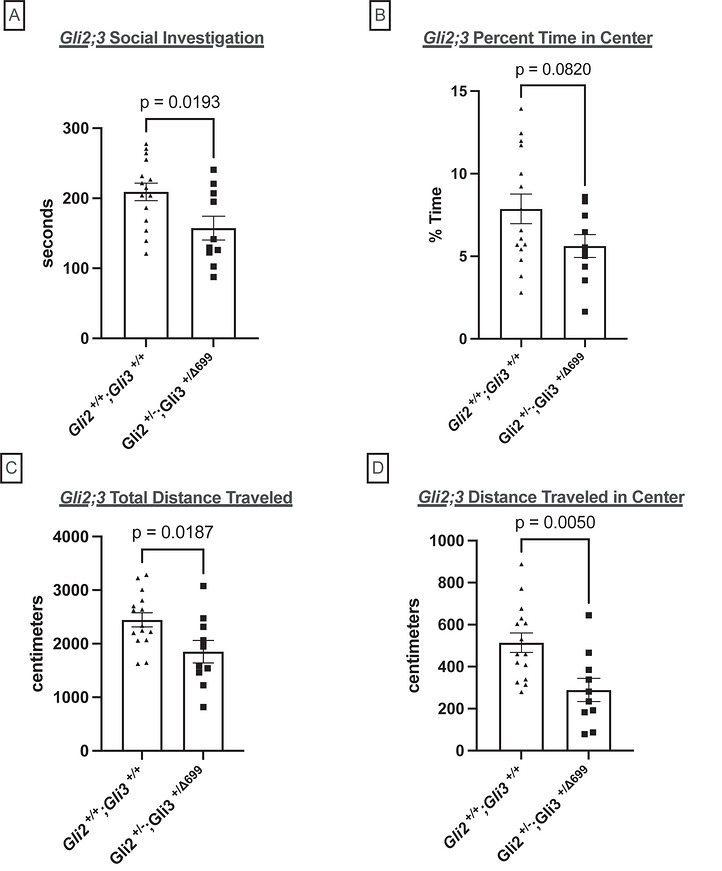
*Gli2^±/−^*; *Gli3^±/𝛥699^* behavioral results. (A) Time spent sniffing, grooming, or following the stimulus mouse within one body length (*F* = 1.237). (B) Percentage of time rodents spent in the center of the open field test (*F* = 2.546). Decreased time indicates increased anxiety‐like behavior. (C) Total distance traveled (*F* = 1.674) and (D) distance traveled in the center (*F* = 1.040) of the open field test. Less distance traveled indicates less exploration.

Juvenile social behavior was significantly reduced in the female double heterozygote *Gli2^+/−^*; *Gli3^+/𝛥699^* mice (Figure 4A). No differences in “jerk and run” behavior were observed. All anxiety‐like behaviors that were altered indicated that female double heterozygote *Gli2^+/−^*; *Gli3^+/𝛥699^* mice experience greater anxiety‐like behavior. This included a decrease in distance traveled in the center (Figure 4C) and a decrease in total distance traveled (Figure 4D). No male *Gli2^+/−^* or *Gli3^+/𝛥699^* mice were viable for behavioral testing.

### 3.4 AGD Morphological Analysis

**FIGURE 5:**
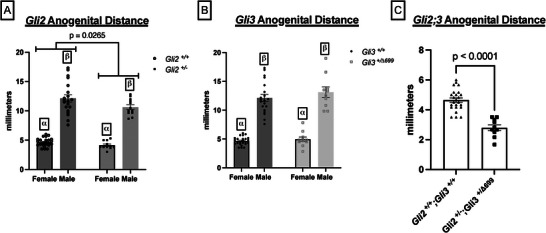
Anogenital distance. (A) *Gli2^+/−^* anogenital distance, [*α*] versus [*β*] represent statistically different groups. (B) *Gli3^+/𝛥699^* anogenital distance, [*α*] versus [*β*] represent statistically different groups. (C) *Gli2^+/−^*; *Gli3^+/𝛥699^* anogenital distance (*F* = 1.458).

AGD was measured as a marker to detect VSCs. Results showed a genotype effect on AGD in *Gli2^+/−^* mice, with a slight reduction in length, regardless of sex (*p* = 0.0265); classical sex differences were also observed ([*α*] vs. [*β*], *p* < 0.0001) (Figure 5A). No differences in AGD were found between heterozygotes and sex matched controls for *Gli3^+/𝛥699^* mice other than classical sex differences (Figure 5B). Additionally, consistent with previous findings (He et al. 2016), female *Gli2^+/−^*; *Gli3^+/𝛥699^* mice exhibited a significant decrease in AGD (Figure 5C).

### 3.5 Sexually Dimorphic Nucleus

**FIGURE 6:**
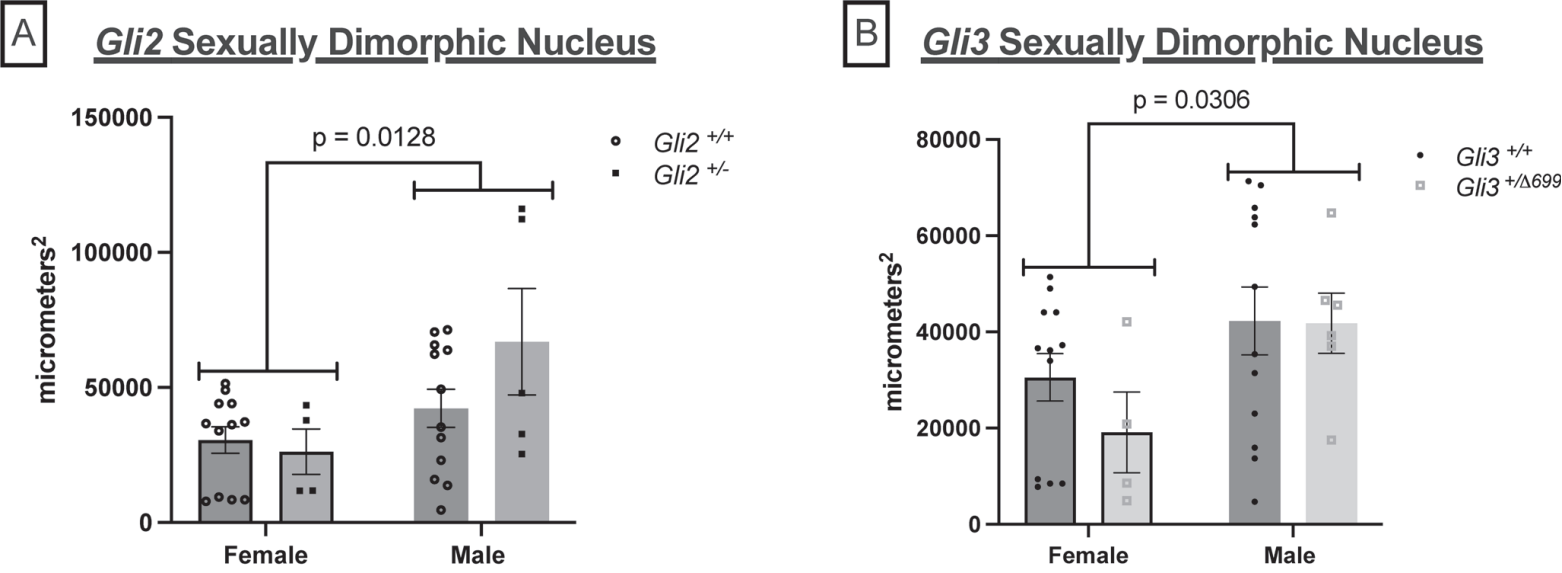
Sexually dimorphic nucleus. Areas of (A) *Gli2^+/−^* and (B) *Gli3^+/𝛥699^* SDN compared to controls.

Although no differences in SDN area were found between heterozygotes and sex matched controls for *Gli2^+/−^* or *Gli3^+/𝛥699^* mice, we did find classical sex differences in SDN size (Figure 6).

### 3.6 Endocrine Assays

**FIGURE 7:**
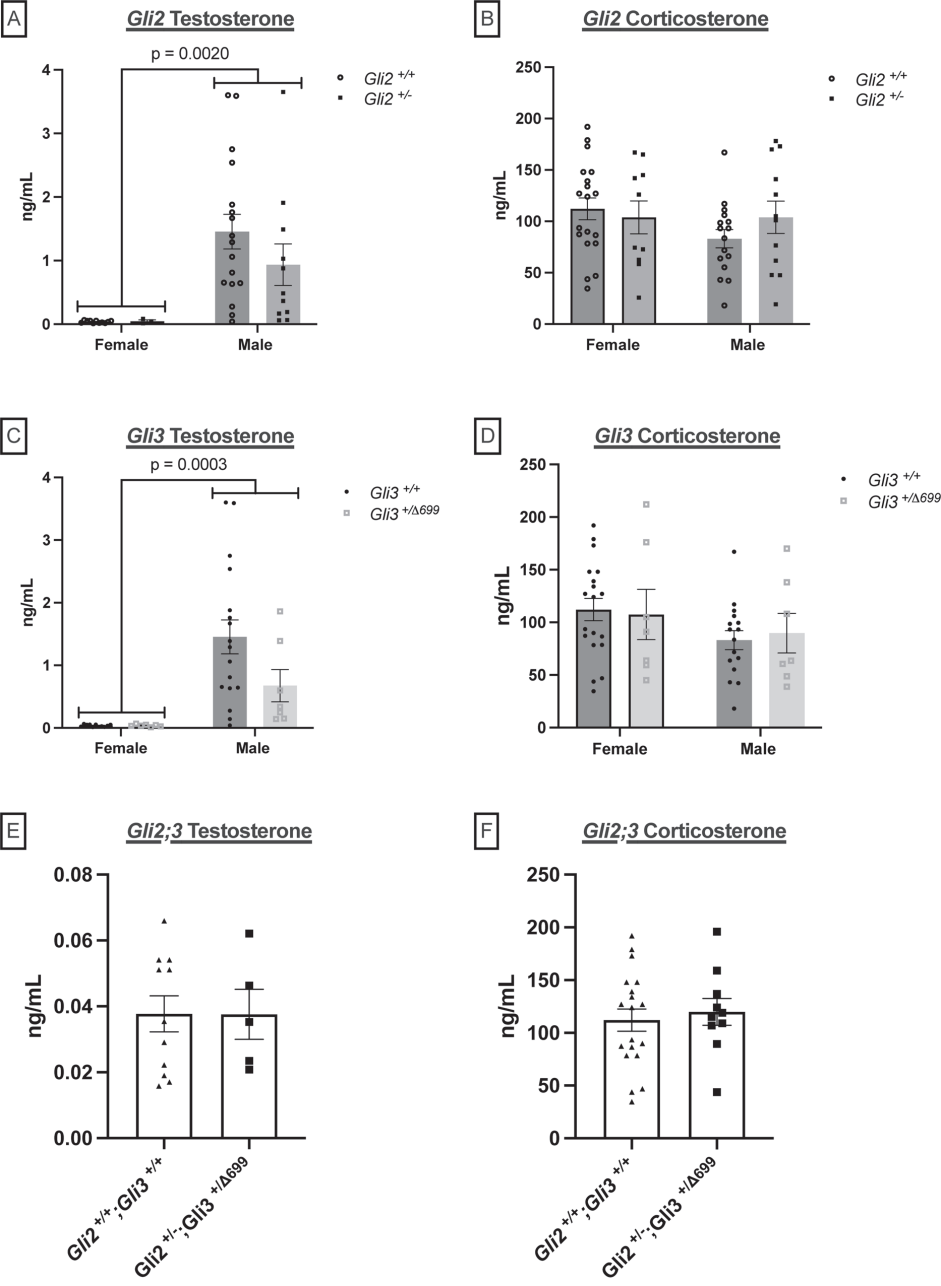
Plasma Steroid Concentrations (A and C) Plasma testosterone of *Gli2^+/−^* and *Gli3^+/𝛥699^* mice, compared to wild type controls; only sex differences observed. (B and D) Plasma corticosterone of *Gli2^+/−^* and *Gli3^+/𝛥699^* mice, compared to wild type controls; no differences observed. (E) Plasma testosterone (*F* = 1.127) and (F) corticosterone (*F* = 1.305) of *Gli2^+/−^*; *Gli3^+/𝛥699^* mice, compared to wild type controls; no differences observed.

No differences in corticosterone were observed in *Gli2^+/−^*, *Gli3^+/𝛥699^*, or *Gli2^+/−^*; *Gli3^+/𝛥699^* in comparison to WT controls (Figure 7B,D,F). Classical sex differences in testosterone were observed in *Gli2^+/−^* and *Gli3^+/𝛥699^* mice (Figure 7A,C). Genotype had no influence on testosterone in *Gli2^+/−^*, *Gli3^+/𝛥699^*, or *Gli2^+/−^*; *Gli3^+/𝛥699^* in comparison to WT controls (Figure 7E).

## 4 Discussion

It is known that GLI2 and GLI3 regulate differentiation of peripheral tissues, neuronal structures, and endocrine organs. The role of GLI2 and GLI3 signaling, as it pertains to juvenile behaviors, is not well understood. Our mutant mice have indicated clear genotypic impacts, with sex‐specific patterns, in behavioral outcomes of *Gli2^+/−^* and female *Gli2^+/−^*; *Gli3^+/𝛥699^* mutant mice. Regardless of sex *Gli2^+/−^* mice showed reduced exploration, whereas male *Gli2^+/−^* mice showed increased non‐locomotive beam break and time spent in the center of the open field test. Female *Gli2^+/−^* and *Gli3^+/𝛥699^* mutant mice showed increased anxiety‐like phenotypes in the open field test and reduced social interaction in the social investigation test. In our testing paradigms, we have reported no behavioral differences in *Gli3^+/𝛥699^* mice. Additionally, we assayed sexually differentiated and endocrine‐sensitive features that are influenced by GLI‐ and hormonally‐regulated pathways: plasma testosterone, plasma corticosterone, AGD, and SDN. Genotypic reductions in AGD were observed in *Gli2^+/−^* mice and female *Gli2^+/−^* and *Gli3^+/𝛥699^* mice, but not *Gli3^+/𝛥699^* mice. No genotypic variations were observed in corticosterone, testosterone, or areas of SDN at euthanasia. Importantly, there was a lack of statistical power in the number of male *Gli2^+/−^* and *Gli3^+/𝛥699^* mice due to decreased viability, so no behavioral or physiological analysis was conducted. Taken together, our data indicate GLI2 and GLI3 signaling deficits connect urogenital development and behavioral outcomes that may be independent of endocrine action.

### 4.1 Social Investigation and Open Field Behavioral Tests

The social interaction test was used to examine variations in social development. We find no significant differences in the social investigation test for *Gli2^+/−^* nor *Gli3^+/𝛥699^* mice. In contrast, female *Gli2^+/−^* and *Gli3^+/𝛥699^* mice exhibited significant decreases in social investigation based on the observation that they spent less time investigating the novel mouse. Lower levels of sociability may be an indicator of social anhedonia, a common symptom across human mood disorders. As neither *Gli2^+/−^* nor *Gli3^+/𝛥699^* modulation alone resulted in a divergent behavioral phenotype, these data on social investigation suggest a compounding role for GLI2 and GLI3 together in the modulation of juvenile social interactions. Curiously, we find a trending reduction in social investigation within *Gli2^+/−^* mice (Figure 2A, *p* = 0.0771) which may indicate some subtle disruption in social behavior that may be augmented when GLI3 signaling is also disrupted.

**FIGURE 8:**
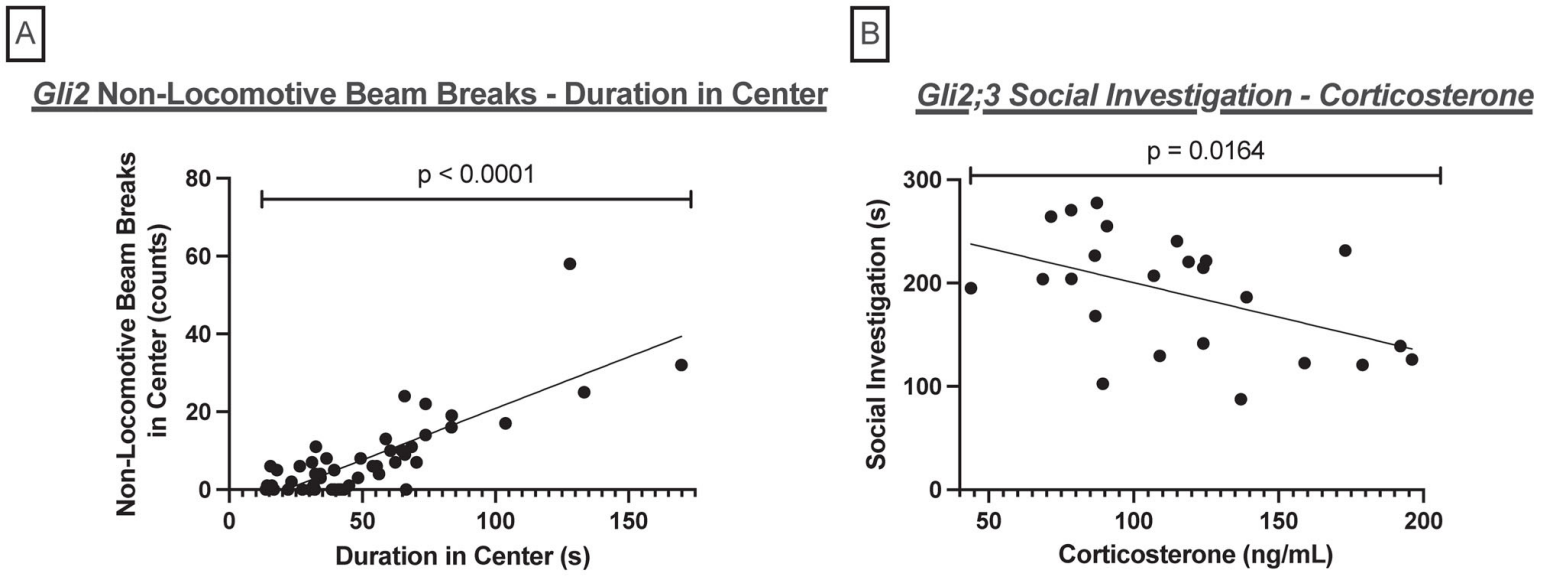
Gli behavior correlations. (A) Male *Gli2^+/−^* mice that spend more time in the center of the open field test show a positive correlation with increased time spent in non‐locomotive beam break activity in the center. (B) Female *Gli2^+/−^* and *Gli3^+/𝛥699^* mice with higher levels of corticosterone show a negative correlation with time spent sniffing, grooming, or following the stimulus mouse within one body length in the social investigation test.

In the open field test, male and female *Gli2^+/−^* mice and female *Gli2^+/−^* and *Gli3^+/𝛥699^* mice exhibited genotypic‐derived behavioral differences. Female *Gli2^+/−^* and *Gli3^+/𝛥699^* mice were observed traveling significantly less in the center, and during the entirety of the open field test, suggesting an increase in anxiety‐like behavior. However, the percentage of time spent in the center of the open field test was only a slight reduction (Figure 4B, *p* = 0.0820). *Gli2^+/−^* mice displayed reduced vertical activity in the center of the open field. Vertical activity has been associated with novel environment nonsocial exploration (Bailey and Crawley 2009), suggesting a disruption in investigative behavior or reduced exploratory drive. This reduction in exploration parallels the subtle disruption in social behavior observed in the social investigation test. Additional observations in the open field test revealed male *Gli2^+/−^* mice spent a significantly longer time in the center of the open field arena than their WT sex‐matched controls. This is a sign of reduced anxiety‐like behavior. Importantly, further correlational analysis revealed that the increase in time was spent engaging in increased non‐locomotive beam break activities (Figure 8A, *p* < 0.0001, *F* = 82.50). Non‐locomotive beam breaks may represent grooming or head bobbing behavior, which are components of stereotypy behaviors, which can occur independent of anxiety‐like behavior and has been reported in a variety of behavioral and neurodevelopmental phenotypes (Mason 1991); however, one report suggests stereotypy is increased in more anxious rodents in the open field (Estanislau et al. 2013). Our data indicate GLI2 modifications influence time spent in the center of the open field, a marker of anxiety‐like behavior, and non‐locomotive beam break behaviors, in only males. Further assessment needs to be done to understand what underlines these significantly different behavioral observations and how they may relate to stereotypy.

Variations in anxiety‐related pathways often show sex‐specific patterns, though these effects can depend on strain and developmental stage. Our mice are on a CD‐1 background, and CD‐1 mice performance in the open field test typically does not differ between sexes in adulthood (Yohn et al. 2019; Makinson et al. 2017). These findings are consistent with the absence of sex differences in our control group. In contrast, our GLI2‐mutant males exhibited decreased anxiety‐like behavior relative to control males, whereas females were unaffected. This indicates a sex‐dependent role of GLI‐mediated signaling in modulating anxiety circuits.

The sex‐specific effect of non‐locomotive beam break behavior may be a result of endogenous estrogenic signaling, which is known to be more pronounced in females than males. For example, *Nrxn1α*, a gene important in synaptic regulation and neural patterning, has been knocked out in mice, which resulted in increased stereotypic behavior (Etherton et al. 2009; Grayton et al. 2013). In other research, estrogen treatment has been shown to partially rescue altered neurogenic phenotypes in models targeting *Nrxn1α* disruption (Willsey et al. 2021). This action was directed via inhibition of SHH signaling. Given that GLI2 functions downstream of SHH, it is possible that our observation of sex differences in non‐locomotive beam break behavior among *Gli2^+/−^* mice may reflect estrogen's ability to buffer SHH–GLI dysregulation. Taking these data together suggests that the *Gli2^±^* has a nuanced relationship with behavior and that GLI2 may regulate multiple neural pathways in a sex‐specific manner. Future research could test if estrogenic treatment could rescue behavioral changes in males.

In our model, a net reduction in HH translational activity results from decreased GLI2 activator signaling coupled with increased GLI3 repressor activity. GLI transcription factors have an intricate balance when activated via HH ligands binding to PTCH1 (Goetz and Anderson 2010). GLI2 predominantly functions as a transcriptional activator, with its stability and nuclear translocation regulated by upstream HH signals through SMO and key kinases such as DYRK2, which phosphorylates GLI2 to release it from the repressor complex suppressor of fused (SUFU) (Yoshida et al. 2024). Conversely, GLI3 largely acts as a transcriptional repressor through proteolytic processing, generating truncated forms that inhibit target gene transcription, in the absence of HH signaling (Ulloa et al. 2007). Importantly, our *𝛥699* model renders the GLI3 protein a constitutive repressor so its continual presence will negatively impact GLI2 activator function. Although direct genetic models targeting GLI modulation are limited, studies of upstream regulators such as SMO reveal parallel behavioral phenotypes to our model: conditional *Smo* knockout induces heightened anxiety‐like behaviors and hyperactivity in rodents (Wang et al. 2022; Zhou et al. 2016), underscoring the essential role of SMO‐mediated GLI activation in neural circuits. Furthermore, stress paradigms reduce hippocampal *Gli2* expression correlating with increased anxiety‐like behavior, effects reversible by interventions that restore *Gli2* levels (Tayyab et al. 2019). Our findings reinforce this mechanistic framework by providing a direct genetic link between altered GLI2 transcriptional activity and anxiety‐like phenotypes, highlighting the critical temporal and molecular control exerted by GLI2/GLI3 balance over both developmental patterning and subsequent behavioral outcomes.

### 4.2 Corticosterone Signaling and Behavior

Given that (1) *Gli3^𝛥699/𝛥699^* double mutants lack adrenal glands (Böse et al. 2002), (2) *Gli2^−/−^* mutants have a reduction in pituitary corticotropes, (3) mosaic mutants (*Gli2^zfd/zfd^*) exhibit pituitary hypoplasia, and even complete absence in pituitary formation (H. L. Park et al. 2000; Wang et al. 2010), and (4) mice with mutations in SHH signaling have disruptions in corticosterone production by P21 (Huang et al. 2010), we assessed for variations in corticosterone levels. Results emphasize, however, that partial *Gli2* and/or *Gli3* modulation does not disrupt corticosterone levels at our euthanasia timepoint. This parallels findings that indicated *Gli3^𝛥699/𝛥699^* double mutants have normal adrenal development (Laufer et al. 2012).

Similarly, *Gli2^+/−^* and *Gli3^+/𝛥699^* female mice exhibited no differences in corticosterone levels; however, we find that higher corticosterone was associated with decreased social investigation (Figure 8B, *p* = 0.0164, *F* = 6.754). Although corticosterone can be a hormonal proxy of stress, it can also be increased in animals with increased arousal or heightened anxiety. Given that the animals are socially isolated before social investigation testing, *Gli2^+/−^* and *Gli3^+/𝛥699^* female mice may be at a higher risk for stress response in these environments. These data could also suggest a difference in corticosterone sensitivity that is not present in *Gli2^+/−^* or *Gli3^+/𝛥699^* mice. With changes in sociability that are correlated with peripheral corticosterone levels, future research should explore glucocorticoid receptor profiles within the *Gli2^+/−^* and *Gli3^+/𝛥699^* mice. No other correlations were found between circulating hormones and behavioral changes.

### 4.3 Testosterone Signaling Verses GLI Independence

It is well known that males have a larger AGD contrasted to females. This sex difference is established primarily due to differences in androgen exposure during perinatal development, although some data indicate that it can be modified by androgen exposure during the juvenile period (Kita et al. 2016; Pakarainen et al. 2005; Dela Cruz et al. 2023). Our current data suggests an influence of GLI2 dosage (*Gli2^+/−^*) on AGD during development. As expected, we documented that males have significantly longer AGDs than females. We also observed a significant genotype effect, independent of sex, indicating a shortening of AGD. This was not mirrored in *Gli3^+/𝛥699^* mutants (Figure 5A). At least during sex differentiation, it seems there is sufficient androgen to promote the increased length consistent with the male AGD; however, GLI2 modulation may blunt this development, regardless of sex. As testosterone is a major contributor to sex differences, including the lengthening of AGD, and *Gli2^zfd/zfd^* and *Gli2^−/−^* double mutants exhibit disruptions in typical pituitary formation and of endocrine hormone production (H. L. Park et al. 2000; Wang et al. 2010), we wanted to examine if testosterone was altered in *Gli2^+/−^* or *Gli3^+/𝛥699^* mice. Although AGD is primarily driven during perinatal development, we found no significant differences in juvenile serum testosterone levels in *Gli2^+/−^* or *Gli3^+/𝛥699^* mice compared to controls. As expected, however, we did confirm classical sex differences in testosterone levels, with males having higher levels than females. These data suggest that *Gli2* haploinsufficiency can affect AGD length, and this difference is maintained even in the presence of typical circulating androgen levels during the juvenile period. Although AGD has been measured in *Gli2^+/−^* mice previously (He et al. 2016), no differences were observed at embryonic day 18.5; however, with increased sample size, investigated at a later developmental date, and comparison of male and female *Gli2^+/−^* mice, we are the first to report this genotype difference. This effect of decreased AGD was further exaggerated in female *Gli2^+/−^*; *Gli3^+/𝛥699^* mice, where AGD was reduced compared to WT controls, but again there were no reported differences in circulating testosterone levels in these animals at euthanasia.

We extended our investigation to another classic marker of perinatal androgen exposure: the SDN of the preoptic area, a brain region typically larger in males as a result of increased locally aromatized androgen exposure. Sex differences in the size of the SDN are known to be driven by perinatal androgen exposure, with females exhibiting greater cellular apoptosis that is maintained into adulthood; therefore, the size of the SDN can be used as a marker for perinatal androgen exposure (Döhler et al. 1984; Gorski et al. 1978, 1980; Amateau and McCarthy 2002). Importantly, neither GLI2 nor GLI3 signaling is known to contribute significantly to hypothalamic development of the preoptic area (Haddad‐Tóvolli et al. 2015), making the SDN a valuable readout of androgen‐mediated, rather than GLI2‐ or GLI3‐mediated, development. Consistent with the framework of endocrine action versus GLI‐mediated signaling, we observed no genotype‐driven differences in SDN volume; however, we did find expected sex differences in SDN volume within both *Gli2^+/−^* and *Gli3^+/𝛥699^* mice (Figure 6). These data further suggest that signaling via aromatized androgens is not significantly altered in early development of the *Gli2^+/−^* or *Gli3^+/𝛥699^* mouse. Importantly, as the double het is lethal in males, we were unable to examine the impact of *Gli2^+/−^* and *Gli3^+/𝛥699^* on SDN morphology.

Together, these data on postnatal AGD, SDN area, and testosterone levels suggest that GLI2, and possibly the combined action of GLI2 and GLI3, influence tissue‐level morphogenesis of androgen‐sensitive features through mechanisms that are at least partly independent of classical androgen signaling. Again, we observed sex‐specific differences in anxiety‐like behavior without corresponding postnatal testosterone changes or alterations in the SDN area. The persistence of these behavioral differences, despite unchanged hormone levels and SDN size, raises the possibility that GLI2 intersects with sex‐dependent developmental pathways beyond traditional androgen‐driven sexual differentiation. These findings point to GLI‐mediated patterning as a potential contributor to sexually differentiated structures and behaviors through pathways that may operate independently of androgen changes, opening new avenues for understanding the genetic modulation of sex‐specific behavior. Our results further suggest that GLI2 may have a broader influence on juvenile behavioral development and modular role on AGD than GLI3 alone, whereas GLI2 and GLI3 together may more strongly affect peripheral anogenital development and behavior. Investigations across more finely resolved prenatal and postnatal time points will be necessary to clarify these relationships.

### 4.4 Integrative Defects in GLI Signaling and Androgen Crosstalk in *Gli2^+/−^*; *Gli3^+/𝛥699^* Male Mice

Although male *Gli2^+/−^* and *Gli3^+/𝛥699^* mice have been previously documented, our attempts to generate a viable cohort revealed profound developmental constraints, leading to an inability to assess behavioral, central, or peripheral phenotypes. This outcome is surprising as it has not been previously reported, but strain shift and environmental factors could account for selecting this negative phenotype. It has been similarly reported that animals with *Gli3^𝛥699/𝛥699^* modulation may have had a strain shift that influenced phenotypes reported in different labs (Böse et al. 2002; Laufer et al. 2012). In our colony, only three male *Gli2^+/−^; Gli3^+/𝛥699^* mice survived into the juvenile period, with only one reaching behavioral testing before succumbing to severe congenital malformations involving the urogenital and peripheral tissues (Supporting Information). The absence of live‐born human cases with dual GLI2 and GLI3 mutations further underscores the developmental indispensability of their combined activity.

These findings suggest that combined partial loss of GLI2 and GLI3 collapses critical HH‐dependent transcriptional networks that coordinate multi‐organ morphogenesis in male. Similarly, the loss‐of‐function mutant *Gli3^XtJ^* mouse (Hui and Joyner 1993) exhibits VSC like characteristics and testicular hormone deficiency (Kothandapani et al. 2020). It is known in these animals that GLI2 and GLI3 act as graded effectors within hedgehog signaling domains that overlap spatially and temporally with androgen receptor (AR) activity in the developing urogenital ridge. With loss of GLI3 activity, morphogen gradient integrations between HH ligands, including Desert and Sonic, negatively affect AR targets controlling Leydig cell differentiation and thus steroidogenesis. Importantly, this steroidogenesis ensures a temporal burst of androgen synthesis necessary for male genital tubercle and anogenital development, with its disruptions in *Gli3^XtJ^* mice leading to the VSC like phenotypes. In our model, there is reduced GLI2 signaling and a Δ699 mutation that facilitates constitutive expression of the GLI3 repressor form, leading to similarly disrupted anogenital development that is more severe. This concurrent double haploinsufficiency compounds a greater disruption of GLI morphogen gradients that flatten HH gradient actions, that singular *Gli2^+/−^* or *Gli3^+/𝛥699^* genotypes are resistant to. With disrupted morphogen gradients, it is likely to lower AR‐driven morphogenetic signaling below viability thresholds, which resulted in our observed malformations that impaired urethral outflow and bladder emptying, promoting early lethality in specifically males. Further, this is not the only sex difference across our cohorts of animals, as sex differences in behavioral outcomes of *Gli2^+/−^* mice were observed. This indicates that androgen–HH feedback circuits modulate GLI dosage sensitivity differently across sexes. The lethality in *Gli2^+/−^*; *Gli3^+/Δ699^* males, and not females, thus exemplifies how crosstalk between HH‐dependent transcriptional regulators and sex different signaling converges on a narrow developmental window, where slight deviations in GLI balance disrupt the integrated morphogenetic program, culminating in male‐specific inviability.

### 4.5 Translation

Mouse models are vital for studying genital anomalies due to conserved developmental pathways, gene regulation, and hormone actions shared with humans (Clarnette et al. 1997; Simmons 2008). They enable controlled investigation of genetic and molecular mechanisms underlying reproductive tract morphogenesis (Cunha et al. 2019; Moon 2006), including external genitalia development (Simmons 2008; Hashimoto et al. 2019; Moon 2006). Critically, rodent models also facilitate exploration of how genetic and environmental contributions to central nervous system‐directed behaviors are related to the same processes involved in peripheral physiological development. Given the association of GLI signaling disruptions with urogenital malformations such as hypospadias and cryptorchidism—and their linkage to neurodevelopmental and psychiatric disorders (Falhammar et al. 2018)—investigating GLI proteins may yield improved diagnostics, prevention, and therapeutics for these conditions. By bridging translational and clinical perspectives, this research has the potential to improve outcomes for individuals with developmental disorders that impact both physical and mental health due to disruptions in GLI signaling.

Our work identifies GLI2 and GLI3 signaling pathways connecting peripheral morphogenesis and social and anxiety‐like behaviors. The *Gli2^+/−^*, *Gli3^+/𝛥699^*, and *Gli2^+/−^; Gli3^+/𝛥699^* mouse models thus offer a platform to probe mechanistic links between VSCs and behavioral phenotypes. Clinically, elucidating the role of hedgehog‐GLI signaling in urogenital and neural development could guide genetic counseling practices and targeted interventions to alleviate mental health burdens in affected individuals. Although current interventions for HH–GLI signaling aberrations are approved for the treatment of various cancers (Mahindroo et al. 2009; Peer et al. 2019), our model provides a platform to actionize these treatments in new ways during early development to optimize developmental outcomes in peripheral and behavioral development. Importantly, these findings are not intended to frame VSC as a pathology requiring correction, but rather to advance understanding of VSC‐associated developmental biology in service of patient‐centered, harm‐reducing clinical care for individuals who experience associated physical or psychological burdens. Again, the use of these mutant mice advances translational understanding with potential to inform genetic counseling practices and therapeutic management of mood disorders linked to developmental disruptions.

Although this study highlights behavioral changes with peripheral morphometric and hormonal measures and examines the SDN of the preoptic area, it acknowledges limitations. Future work will focus on Gli‐dependent neuroanatomical changes in key limbic regions such as the amygdala and hippocampus, which are integral to anxiety‐like and social behaviors. Additionally, larger cohorts and varied social paradigms will help capture subtle, context‐dependent behavioral alterations, including cognitive behaviors that are known to be modulated with *Gli3* mediated dysregulation. These complementary studies aim to provide a more mechanistic, integrated, and holistic understanding of how GLI modulation drives the behavioral phenotypes we report, that may further emphasize how peripheral changes in GLI sensitive tissues connect to brain and behavioral outcomes.

## 5 Conclusion

Our study establishes the first connection of altered juvenile social and anxiety‐like behavior in mice with direct genetic disruptions in GLI signaling. Specifically, decreased GLI2 function modulated anxiety‐like behavior with sex‐specific differences. Additionally, the *Gli2^+/−^*; *Gli3^+/𝛥699^* mutant mouse had negative influences on juvenile social and anxiety‐like behaviors. We have also investigated sexual differentiated tissues that are influenced by GLI regulated and hormonally regulated pathways and have concluded that the observations we report are primarily GLI derived. Finally, these studies provide insight into how some genes that result in genital variations may also regulate neuronal processes related to anxiety‐like behavior and social development. Again, as disruptions to GLI2 and GLI3 signaling pathways occur simultaneously and escalate in severity, alterations in not only the peripheral morphology but also changes in behavioral development are likely to be observed.

## Author Contributions

**Thomas Niepsuj**: writing – original draft, investigation, methodology, validation, visualization, writing – review and editing, software, formal analysis, data curation, conceptualization. **Rithika Nurani**: investigation, writing – review and editing, methodology. **Leykza Carreras–simons**: investigation, methodology, writing – review and editing. **Wade Bushman**: writing – review and editing, conceptualization. **Joan S. Jorgensen**: investigation, conceptualization, supervision, writing – review and editing, writing – original draft. **Walid A. Farhat**: conceptualization, writing – review and editing, investigation, funding acquisition, writing – original draft, supervision, resources. **Anthony P. Auger**: conceptualization, writing – review and editing, investigation, supervision, writing – original draft.

## Ethics Statement

All protocols and procedures involving animals were reviewed and approved by the Institutional Animal Care and Use Committee (IACUC) of the University of Wisconsin (IACUC protocol number: M006295‐R02).

## Conflicts of Interest

The authors declare no conflicts of interest.

## Supporting information



Supplemental Report: PDF of Autopsy report

## Data Availability

Data available on request from authors.
