# Supplementary material for: Social and Anxiety‐Like Behaviors Are Affected in Juvenile Mice With Gli2+/− but Not Gli3+/𝛥699 Genetic Modifications
Source: Brain Behav. 2026 May 31;16(6):e71503. doi: 10.1002/brb3.71503 (PMC13239559; doi:10.1002/brb3.71503)
Supplement: Supplementary file 1 — Supplemental Report: PDF of Autopsy report [file BRB3-16-e71503-s001.pdf]

Three COMPARATIVE PATHOLOGY LABORATORY
Research Animal Resources and Compliance, 302 Enzyme Institute
1710 University Avenue, University of Wisconsin
Madison, WI 53726-4087
Clinical Lab • **Histo Lab** • **FAX 6**

RODENT

GENERAL INFORMATION:
Submission Date: **4-11-2022** Protocol Number:
Direct charge number required for billing: Billing Officer:
College:(CALS, GRAD, SMPH, L&S, SVM) Department: Lab Animal Veterinarian:

LABORATORY INFORMATION:
Investigator: Telephone: Lab Contact Person: Facility Contact:
Email:

ANIMAL INFORMATION:
Species: **mus** Strain/Breed: **Gli2/Gli3 mutants** Bio level: **1**
No.: **3** Age: **DOB 3/24/2022** Sex: **M** ID: Animal Room No.: **600**
Specimen Submitted: **Whole bodies**
Live Dead Euthanized **X** Method and drug used **Not provided**
Date & time of Death: **12:55 pm April 11th 2022.**
Experimental procedures, drugs, diet and/or transgene/mutation: **Gli2/Gli3 double heterozygous mutations**

Preliminary Findings

Confidential Study Data

History: A few of the pups appeared runted to staff at sampling on the previous Friday (April 8th). This is the parents' first litter. There are 4 other breeding pairs; staff has seen runty pups before but not like these. There are 13 total pups in this litter. The lab has also noticed low survival in double heterozygous mice. The lab requests necropsy examination of these three runty pups to look for factors that may relate to low survival. The lab requests that the brains be removed and placed in formalin to be picked up by the lab.

Mouse 705930 = R ear punch
Mouse 705931 = L ear punch
Mouse 705940 = RRL ear punch

GROSS NECROPSY

Receipt: All 3 mice are received in a bag labeled "24887 4/11/22"

MOUSE A (705931)

External Findings – Received is the body of a juvenile white male mouse weighing 4.0090g with a body condition score of 2/5. The hair is moderately disarrayed. The left pinna has a semicircular punch in the caudal margin. The eyes are squinted half-closed. The head is not appreciably domed. External ear canals and nares are clean. The incisor teeth occlude appropriately. The oral mucous membranes are medium pink. The limbs palpate within normal limits. All fingers and toes are present in appropriate numbers with nails. The abdomen palpates soft. There is a small amount of dried yellow material on the perineal region and the medial surfaces of the hocks. There is not a grossly identifiable anal opening.

Integument and Subq – The subcutis is moist. There is minimal subcutaneous adipose tissue; it is most evident in the cervical subcutis.

Cardiovascular – The heart is externally red-brown. The left ventricular free wall (LVFW) is 1mm thick and the right ventricular free wall (RVFW) is 0.5mm thick (RVFW:LVFW ratio of 1:2; within normal limits).

Respiratory – The diaphragm is appropriately concave. There is appropriate negative pressure in the thoracic cavity. The caudal 30% of the left lung lobe is collapsed and maroon; the remainder is soft and light pink. The accessory lobe is 95% maroon and the remaining right lung lobes are approximately 50% maroon. The remainder of the right lung tissue is soft and light to medium pink.

Digestive – The salivary glands are medium pink and symmetrical. The esophagus is uniform and has a light pink serosa. Abdominal serosal surfaces are moist. The stomach has a pale tan serosa in the squamous portion and a tan serosa in the glandular region. The stomach contains a small amount of soft moist white material and has a smooth translucent light tan mucosa. The duodenum has a light pink serosa and contains a small amount of mucoid tan material. The jejunum is externally soft and has an orange serosa. The jejunum contains a small to moderate amount of thick yellow tan material. The ileum has a light tan serosa. The cecum has a tan yellow serosa, contains a small amount of semi-formed tan yellow material, and has a translucent mucosa. The colon has a tan yellow serosa. The colon at the rostral edge of the pelvic canal is 4 x 2 mm in cross section. The proximal colon contains a small amount of pale tan mucoid material. The mid- to distal colon contains a large amount of semi-formed yellow tan material. The rectum narrows to less than 1 mm in external diameter. A 1-mm diameter probe can be passed into the lumen of the distal rectum but cannot be passed through to the skin surface. There is no grossly apparent anus, and feces cannot be manually expressed through any observed anal openings. The liver is medium red brown with sharp edges. The pancreas is light pink and smooth. There is minimal to absent mesenteric and omental adipose tissue.

Lymphohematopoietic – The cervical lymph nodes are indistinct. There is a small to moderate amount of thymic tissue. The spleen is medium pink and has sharp edges. It measures 9 mm long.

Uro-Genital – The kidneys are similar in size with medium red brown cortices and good corticomedullary distinction. The ureters are symmetrical and light pink. They are nondilated and insert appropriately at the trigone. The urinary bladder is contracted and has a light tan serosa. The testicles and epididymides are symmetrical and pale tan. The seminal vesicles are symmetrical and light yellow. The preputial glands are symmetrical and pale tan. The ventral aspect of the prepuce is slightly separated on midline over a region estimated at approximately 1.5 x 2 mm and beginning at the preputial orifice; it is not clear grossly (or via dissecting microscope) whether the penis is involved.

Endocrine – The adrenal glands are symmetrical and light pink; each is approximately 1.5 mm in diameter. The thyroid glands are symmetrical, light pink, and grossly unremarkable. The pituitary gland is unremarkable in size and location grossly.

Central Nervous System – Upon disarticulating the head, a portion of caudal cerebellum that measures 2mm in the dorsoventral plane is visible in the foramen magnum. The brain is otherwise symmetrical and light pink.

Peripheral Nervous System – There are no significant gross abnormalities.

Musculoskeletal – There are no significant gross abnormalities.

Mouse A:

**Body Weight (g)**	4.0090g	
**Organ**	**Weight (g)**	**%BW**
**Brain**	*	
**Spleen**	0.0072g	0.180%
**Liver**	0.1953g	4.872%
**Left Kidney**	0.0396g	0.988%
**Right Kidney**	0.0442g	1.103%
**Heart**	0.0429g	1.070%

*Brain is not weighed due to autolysis/tissue fragility

Preliminary Findings

Preliminary Findings

MOUSE B (705940)

External Findings – Examined is the body of a juvenile male white mouse weighing 5.189g with a body condition score of 2/5. There is a semicircular punch in the caudal margin of the left ear. In the right ear there is a similar punch in the rostral margin near the tip and another punch in the caudal margin near the tip. The head is not appreciably domed. The haircoat is slightly disarrayed. The right eye is moderately squinted. The corneas are slightly dry and clear, and the nares and external ear canals are clean. The incisor teeth occlude appropriately. Oral mucous membranes are medium pink. The limbs palpate within normal limits. Appropriate numbers of digits are present with nails. The abdomen palpates soft. There is a small amount of dry yellow material surrounding the perineal region and on the hind limbs. There is no externally visible anal opening.

Integument and Subq – The subcutis is moist, and there is minimal subcutaneous adipose tissue.

Cardiovascular – The epicardial surfaces of the ventricles are medium pink. The auricles are externally maroon and similar in size. The left ventricular free wall (LVFW) is 1mm thick, and the right ventricular free wall is 0.5mm thick (RVFW:LVFW ratio of 1:2; within normal limits).

Respiratory – The diaphragm is appropriately concave and there is appropriate negative pressure in the thoracic cavity. The right cranial lung lobe is maroon. The right middle lobe is 60% maroon and the right caudal lobe is 10% maroon multifocally. The caudal 70% of the left lobe is maroon. The accessory lobe is maroon. The remainder of the lung tissue is soft and light pink.

Digestive – The salivary glands are symmetrical and light pink. The esophagus is uniform with a light pink serosa. Serosal surfaces of the abdomen are moist. The stomach has a pale tan serosa and contains a small amount of soft to pasty white material and hair, including a small aggregate of hair at the pylorus. The gastric mucosa is smooth and light tan. The duodenum has a light pink serosa and contains a small amount of tan mucoid material. The jejunum has a light tan yellow serosa and contains a small amount of tan yellow thick fluid. The ileum has a light tan pink serosa and has similar contents to those in the jejunum. The cecum has a tan yellow serosa and contains a moderate amount of pasty yellow tan material. The colon has a tan yellow serosa and contains a large amount of semi-formed tan yellow material. The colon at the rostral edge of the pelvic canal is approximately 4x4 mm in cross section. The rectum narrows to less than 1mm in external diameter. A 1mm-diameter probe can be passed in the lumen of the rectum but does not communicate with the skin. There is no grossly evident anal opening. The liver is light to medium red brown with sharp edges. The pancreas is light pink and smooth. There is minimal to absent mesenteric and omental adipose tissue.

Lymphohematopoietic – There is a moderate amount of pale pink thymic tissue. The spleen is medium pink, has sharp edges, and measures 1cm long.

Uro-Genital – The kidneys are similar in size and have red brown cortices. The kidneys have good corticomedullary distinction, though the left kidney on longitudinal cut section has two apparently separate medullary pyramids (vs. sectioning artefact; see comment). The ureters are similar to those of Mouse A. The urinary bladder has a light tan serosa and is contracted. The testicles and epididymides are similar to those of Mouse A. The preputial glands are also similar to those of Mouse A.

Endocrine – The thyroid and adrenal glands are similar to those of Mouse A. The pituitary gland is similar to that of Mouse A.

Central Nervous System – Upon disarticulation of the atlantooccipital joint, a portion of caudal cerebellar vermis measuring 1.5mm in the dorsoventral plane is within the foramen magnum. The brain is otherwise symmetrical and light pink.

Peripheral Nervous System – There are no significant gross abnormalities.

Musculoskeletal – There are no significant gross abnormalities.

Mouse B:

Body Weight (g)	5.189g	
**Organ**	**Weight (g)**	**%BW**
**Brain**	*	
**Spleen**	0.012g	0.231%
**Liver**	0.291g	5.608%
**Left Kidney**	0.061g	1.176%
**Right Kidney**	0.048g	0.925%
**Heart**	0.057g	1.098%

*Brain is not weighed due to autolysis/tissue fragility

<u>**MOUSE C (705930)**</u>

External Findings – Examined is the body of a juvenile male white mouse weighing 5.4873g with a body condition score of 2/5. The right ear has a semicircular punch at the tip. The left eye is squinted shut, and both corneas are clear. The external ear canals and nares are clean. The incisor teeth occlude appropriately. Oral mucous membranes are medium pink. The limbs palpate within normal limits. Appropriate numbers of digits are present with nails. The abdomen palpates soft. The haircoat is slightly disarrayed. There is no externally visible anal opening.

Integument and Subq – The subcutis is moist and there is minimal subcutaneous adipose tissue.

Cardiovascular – The epicardial surfaces of both ventricles and the right atrium are light red brown; the left atrium is maroon. The left ventricular free wall (LVFW) is 1mm thick and the right ventricular free wall (RVFW) is 0.33mm thick (RVFW:LVFW ratio of 1:3; within normal limits).

Respiratory – The middle 70% of the left lung lobe is maroon, as is the entire right cranial lobe and 70% of the right caudal and accessory lobes. The remainder of the lung tissue is soft and light pink.

Digestive – The salivary glands are symmetrical and light pink. The esophagus is uniform and has a light pink serosa. Abdominal serosal surfaces are moist. Due to increased intestinal tissue fragility (suspect autolysis) there is artifactual leakage of contents into the abdominal cavity with gentle manipulation at necropsy; there is no gross evidence of associated fibrin accumulation. The stomach has a pale tan to light pink serosa and contains a small amount of soft white material and hair. The gastric mucosa is light tan pink. The duodenum has a light pink serosa and contains a small to moderate amount of pale tan mucoid material. The jejunum has a tan yellow serosa. The jejunum and ileum both contain a large amount of soft yellow tan material. The cecum has a tan yellow serosa. The cecum is markedly distended with a large amount of soft to thick fluid yellow tan material. The colon has a light pink to tan yellow serosa and contains a large amount of semi-formed yellow tan material. The colon at the level of the rostral edge of the pelvic canal is approximately 2 x 2mm in cross section. A 1mm-diameter probe can be passed through the lumen of the rectum but does not extend to the skin surface; an anal opening is not identified grossly. The pancreas is light pink and smooth. The liver is medium red brown with sharp edges.

Lymphohematopoietic – The spleen is light pink, has sharp edges, and is 9mm long. The thymus is similar to those of Mice A and B.

Uro-Genital – The kidneys are similar in size with medium pink cortices. The kidneys are soft (interpreted as autolysis) but have good corticomedullary distinction. The ureters are similar to those of Mice A and B. The urinary bladder is flaccid, empty, and has a pale pink serosa. The testicles, epididymides, and preputial glands are similar to those of Mice A and B.

Endocrine – The thyroid and adrenal glands are similar to those of Mice A and B. The pituitary gland is pink and grossly unremarkable.

Preliminary Findings

Central Nervous System – Upon disarticulating the atlantooccipital joint, a portion of caudal cerebellum measuring 1mm in the dorsoventral plane is visible at the foramen magnum. The brain is otherwise symmetrical and light tan pink.

Peripheral Nervous System – There are no significant gross abnormalities.

Musculoskeletal – There are no significant gross abnormalities.

Mouse C:

**Body Weight (g)**	5.4873g	
**Organ**	**Weight (g)**	**%BW**
**Brain**	*	
**Spleen**	0.0117g	0.213%
**Liver**	0.300g	5.467%
**Left Kidney**	0.050g	0.911%
**Right Kidney**	0.046g	0.838%
**Heart**	0.038g	0.693%

*Brain is not weighed due to autolysis/tissue fragility

Tentative Diagnoses:

Mouse A:

1. Digestive system:
   a. Imperforate anus (anorectal malformation)
   b. Mid to distal colon: Subjectively moderately increased contents (suspect secondary to anorectal malformation)

2. Urogenital system:
   a. Prepuce: Mild to moderate locally extensive ventral midline defect (see comment)

3. Respiratory system:
   a. Moderate to marked multifocal to coalescing maroon discoloration (congestion vs. hyperemia vs. inflammation vs. postmortem change vs. artefact of euthanasia (see comment) vs. hemorrhage vs. other)

4. Nervous system:
   a. Brain: Moderate cerebellar herniation vs. postmortem softening vs. other

Mouse B:

1. Digestive system:
   a. Imperforate anus (anorectal malformation)
   b. Mid to distal colon: Subjectively moderately to markedly increased contents (suspect secondary to anorectal malformation)

2. Urogenital system:
   a. Left kidney: renal medullary malformation vs. sectioning artefact vs. other (see comment)

3. Respiratory system:
   a. Moderate to marked multifocal to coalescing maroon discoloration (congestion vs. hyperemia vs. inflammation vs. postmortem change vs. artefact of euthanasia (see comment) vs. hemorrhage vs. other)

Preliminary Findings

4. Nervous system:
   a. Brain: Moderate cerebellar herniation vs. postmortem softening vs. other

Mouse C:

1. Digestive system:
   a. Imperforate anus (anorectal malformation)
   b. Jejunum, ileum, cecum, and colon: Subjectively markedly increased contents (suspect secondary to anorectal malformation)

2. Respiratory system:
   a. Moderate to marked multifocal to coalescing maroon discoloration (congestion vs. hyperemia vs. inflammation vs. postmortem change vs. artefact of euthanasia (see comment) vs. hemorrhage vs. other)

3. Nervous system:
   a. Brain: Mild cerebellar herniation vs. postmortem softening vs. other

Pending Lab Procedures: Histopathology

Completed Lab Procedures: N/A

Comment: Possible causes of runting in these three pups are suspected to include imperforate anus, which is incompatible with long term survival; however, if these are global double heterozygous mutations, it is also possible that other systems may be affected in ways not necessarily grossly evident, and that these could also contribute to runting. Accumulation of relatively larger quantities of colonic contents than expected is consistent with inability to pass feces. Imperforate anus is a documented effect of some types of mutations in Gli2/3 (He et al 2016), but the mutations in this case are not specified.

The prepuce of Mouse A exhibits a locally extensive defect/division on the ventral surface extending from the preputial orifice. In consideration of known effects of Gli2 and Gli3 mutations on disorders of sexual differentiation (He et al 2016), it is possible that this may be related to genotype.

The left kidney of Mouse B on cut section has an unusual medullary appearance, though an artefact of gross sectioning plane cannot be ruled out. Histopathology is pending.

The method of euthanasia was not specified in these mice. Euthanasia with carbon dioxide can in some cases result in artefactual congestion and/or hemorrhages in the lungs; if this is the method of euthanasia used in this case, it may contribute to observed pulmonary discoloration. Other potential factors are postmortem change or antemortem changes such as congestion, hyperemia, hemorrhage, inflammation, or other processes.

In many cases, herniation of the cerebellum into the foramen magnum suggests an intracranial mass effect such as edema, inflammation, neoplasia, etc. However, in some cases, softening of the brain due to postmortem change may also contribute to this finding. Histologic examination can aid in evaluation for evidence of true antemortem change such as cerebellar Purkinje cell necrosis or loss.

One potential pathogenesis to consider in this case is that inability to pass feces could theoretically increase the possibility of translocation of digestive flora from relatively static fecal material through the intestinal mucosa. Bacteremia, endotoxemia, and sepsis are potential causes of edema, inflammation, and/or hemorrhage in a variety of tissues. Histopathology is pending and will include further evaluation of genital and lower digestive structures.

References:

He, Fei et al. "Adult Gli2$^{+/-}$;Gli3$^{delta699/+}$ Male and Female Mice Display a Spectrum of Genital Malformation". PLoS ONE. 2016. 11(11): e0165958. doi:10.1371/journal.pone.0165958

Preliminary Findings

HISTOPATHOLOGY

Mouse A:

Slide a: Salivary glands, superficial cervical lymph nodes: thymus, spleen, adrenal glands, testicles, lung, tongue, trachea, tracheobronchial lymph nodes, esophagus, thyroid glands:

Salivary glands: There are no significant histologic lesions (NSL).

Superficial cervical lymph node: There are moderate to large quantities of cortical lymphoid tissue. Endothelial cells lining blood vessels in the sampled lymph node are plump (reactive). Small numbers of macrophages infiltrate the sinuses.

Thymus: There are small to moderate quantities of thymic lymphoid tissue. NSL.

Spleen: There are moderate to large quantities of white pulp; periarteriolar lymphoid sheaths often blend together. The red pulp contains small to moderate numbers of myeloid and erythroid precursor cells, neutrophils, and megakaryocytes (extramedullary hematopoiesis).

Testicles and epididymides: Bilaterally, seminiferous tubules in the testes occasionally contain intraluminal binucleate or trinucleate spermatocytes (degeneration). The epididymides are within normal limits.

Lung: NSL.

Tongue: NSL.

Trachea: NSL.

Tracheobronchial lymph nodes: There are small to moderate quantities of cortical lymphoid tissue. Small numbers of macrophages are present within sinuses.

Esophagus: There are small to moderate numbers of bacterial cocci in the superficial keratin of the mucosa. The lumen contains rare erythrocytes.

Thyroid glands: One thyroid gland is sampled in the examined section. Follicles vary in diameter by approximately 6X, are lined with cuboidal to attenuated epithelium, and contain brightly eosinophilic colloid. NSL.

Slide b: Stomach, perineum, duodenum, pancreas, kidneys, liver, heart:

Perineum: DEEPERS NEEDED

Duodenum: The duodenum is markedly autolyzed in some areas, but villi are preserved in a small locally extensive region. NSL.

Pancreas: The pancreas is moderately autolyzed, but regionally preserved. Rare acinar cells have clear moderatey=ly distinct cytoplasmic vacuoles.

Kidneys: There is mild loss of differential staining in cortical regions (interpreted as autolysis) bilaterally.

Liver: The liver is regionally mildly autolyzed. NSL.

Heart: NSL.

Preliminary Findings

Slide c: Jejunum, stomach, ileocecocolic junction, colon, urinary bladder, rectum, suspect accessory sex glands (see comment):

Jejunum: Examined sections are markedly autolyzed; basic mural architecture is within normal limits, including scattered GALT nodules.

Stomach: Small to moderate numbers of scattered bacilli and short rods are closely associated with the squamous mucosal surface; there is no associated inflammation. The glandular mucosa is mildly to moderately autolyzed. The gastric lumen contains small numbers of round protozoal organisms measuring approximately 15-25 um in diameter with vacuolated cytoplasm and up to 4 nuclei (consistent with Entamoeba species).

Ileum: The ileum is markedly autolyzed; the base of glands is preserved in a few regions. NSL.

Cecum: The cecal mucosa is mildly to moderately autolyzed. There are a few small to moderately sized gastrointestinal associated lymphoid tissue (GALT) nodules, which are within normal limits.

Colon: The colon is moderately autolyzed, with complete loss of mucosa in some regions and relative preservation in others. There are a few small and one moderately sized GALT nodules; these are within normal limits. A single round protozoal organism similar to those described in the stomach is within the colonic lumen.

Urinary bladder: NSL.

Rectum: The mucosa is moderately to markedly autolyzed; the base of glands is not preserved, but basic mural architecture is within normal limits.

Suspect accessory sex glands (see comment): Adjacent to the urinary bladder in the examined section there are multiple tubular profiles lined by stratified cuboidal to pseudostratified columnar epithelium. Lumina of these tubules occasionally contain variable numbers of sloughed epithelial cells.

Mouse B:
Slide a: Salivary glands, superficial cervical lymph nodes, thymus, spleen, adrenal glands, testicles and epididymides, tongue, larynx, trachea, tracheobronchial lymph node, esophagus, lungs, heart, thyroid glands:
a: testicles have a few multinucleated cells (dysplasia?!)
Thymic lymphocytolysis, foamy macrophage infiltration
epididymal tubules have small numbers of neutrophils, bilaterally.

Salivary glands: NSL.

Superficial cervical lymph nodes: There are moderate quantities of cortical lymphoid tissue. Endothelial cells are large and plump (suspect reactive) similar to those described in Mouse A. Subcapsular sinuses occasionally contain small numbers of macrophages.

Thymus: There are moderate quantities of lymphoid tissue. There is marked, often peripheral, karyorrhectic debris (lymphocytolysis) accompanied by moderate to large numbers of tingible body macrophages and foamy macrophages.

A small portion of well-differentiated thyroid glandular tissue is sampled with the thymus (ectopic thyroid tissue).

Spleen: There are moderate to large quantities of white pulp; periarteriolar lymphoid sheaths often blend together. The red pulp contains small to moderate numbers of myeloid and erythroid precursor cells and megakaryocytes (extramedullary hematopoiesis).

Adrenal glands:

Testicles and epididymides:

Preliminary Findings

Tongue: NSL.

Larynx: NSL.

Trachea: NSL.

Tracheobronchial lymph node: There are moderate quantities of cortical lymphoid tissue. Endothelial cells are often plump and oval as described in the superficial cervical lymph nodes above. There are small numbers of macrophages in the subcapsular sinuses.

Esophagus:

Lungs:

Heart:

Thyroid glands: Bilaterally the thyroid glands have follicles lined by predominately cuboidal to occasionally attenuated epithelium. Follicles vary in diameter by 3-4X and contain brightly eosinophilic colloid.

Mouse C:
Slide a: Salivary glands, testicles, epididymides, adrenal glands, spleen, heart, lungs, trachea, esophagus, larynx, tongue, thyroid glands:
a: moderate to marked thymic lymphocytolysis
testicles: occasional multinucleated cells.

Salivary glands:

Testicles and epididymides:

Adrenal glands:

Spleen:

Heart:

Lungs:

Trachea:

Esophagus:

Larynx:

Tongue:

Thyroid glands:

Slide b: Stomach, duodenum, pancreas, kidneys, ureters, liver:

Stomach:

Duodenum:

Preliminary Findings

Pancreas:

Kidneys:

Ureters:

Liver:

Slide c: Rectum, jejunum, ileum, cecum, colon:

Rectum:

Jejunum:

Ileum:

Cecum:

Colon:

*Get serial sections of rectum/anal region in all three

Comment:

Multinucleation of spermatocytes in the testes can suggest a degenerative process. The cause is not clear histologically.

Preliminary Findings
